# Response to genomic selection: The Bulmer effect and the potential of genomic selection when the number of phenotypic records is limiting

**DOI:** 10.1186/1297-9686-44-26

**Published:** 2012-08-03

**Authors:** Elizabeth M Van Grevenhof, Johan AM Van Arendonk, Piter Bijma

**Affiliations:** 1 Animal Breeding and Genomics Centre, Wageningen University, P.B. 338, Wageningen, The Netherlands

## Abstract

**Background:**

Over the last ten years, genomic selection has developed enormously. Simulations and results on real data suggest that breeding values can be predicted with high accuracy using genetic markers alone. However, to reach high accuracies, large reference populations are needed. In many livestock populations or even species, such populations cannot be established when traits are difficult or expensive to record, or when the population size is small. The value of genomic selection is then questionable.

**Methods:**

In this study, we compare traditional breeding schemes based on own performance or progeny information to genomic selection schemes, for which the number of phenotypic records is limiting. Deterministic simulations were performed using selection index theory. Our focus was on the equilibrium response obtained after a few generations of selection. Therefore, we first investigated the magnitude of the Bulmer effect with genomic selection.

**Results:**

Results showed that the reduction in response due to the Bulmer effect is the same for genomic selection as for selection based on traditional BLUP estimated breeding values, and is independent of the accuracy of selection. The reduction in response with genomic selection is greater than with selection based directly on phenotypes without the use of pedigree information, such as mass selection. To maximize the accuracy of genomic estimated breeding values when the number of phenotypic records is limiting, the same individuals should be phenotyped and genotyped, rather than genotyping parents and phenotyping their progeny. When the generation interval cannot be reduced with genomic selection, large reference populations are required to obtain a similar response to that with selection based on BLUP estimated breeding values based on own performance or progeny information. However, when a genomic selection scheme has a moderate decrease in generation interval, relatively small reference population sizes are needed to obtain a similar response to that with selection on traditional BLUP estimated breeding values.

**Conclusions:**

When the trait of interest cannot be recorded on the selection candidate, genomic selection schemes are very attractive even when the number of phenotypic records is limited, because traditional breeding requires progeny testing schemes with long generation intervals in those cases.

## Background

Genomic selection (GS) is a variant of marker-assisted selection in which genetic markers covering the whole genome are used so that all quantitative trait loci (QTL) are in linkage disequilibrium with at least one marker [1]. Simulation results and practical data in dairy cattle suggest that breeding values can be predicted with high accuracy using genetic markers alone [2,3]. Since the introduction of the idea by Meuwissen et al. [4] ten years ago, there has been a number of developments, including the implementation of GS in dairy cattle breeding [3]. Until recently, the major limitation to implement GS was the large number of markers required and the cost of genotyping these markers. Both these limitations have now been overcome in most livestock species, following the sequencing of the genomes and the subsequent availability of high-density SNP chips [1]. It is now feasible to meet the requirements for the implementation of GS in breeding programs. In fact, after deriving a prediction equation from a reference population that uses markers and phenotypes as input and predicts breeding values as output, there is in principle no need to record phenotypes of the candidates for the selection. Thus, GS can potentially cut costs for producing and testing potential breeding animals considerably. Moreover, GS can have a large impact on breeding programs for many livestock species as it can shorten generation intervals, which is of special importance in long-lived species such as dairy cattle and horses, or when trait values become available late in life or on progeny only. This is important, as genotyping can be applied to new-born animals or even embryos [5], and because of the reduced need for progeny testing.

A limitation of GS, however, is that large reference populations are needed to obtain high accuracies of estimated breeding values (EBV). When the size of the reference population increases, the accuracy of EBV can reach high values, approaching 0.8 to 1.0 [4,6,7]. Reference population sizes used in simulations sometimes even exceed 100 000 animals [8] but in practise, reference populations are in some cases limited to less than 1000 animals [9]. In many livestock populations and some livestock species, creation of large reference populations is not very feasible for many traits, especially when phenotypes are difficult or expensive to record, such as methane emission in cattle or traits related to disease resistance. If large reference populations cannot be obtained, GS will reach relatively low accuracies, and may yield no or relatively little additional response compared to traditional selection on EBV based on phenotypic information. This applies particularly to populations with a large historical effective size, and to traits that are determined by many genes, which is common in livestock [10,11]. The more genes involved, the smaller the effect of individual genes, and the larger the reference population needed to reach a certain accuracy [12]. For those reasons, it is important to investigate when GS offers advantages over traditional selection in cases in which the size of the reference population is limited.

In this study, we compared response to GS with response to selection on BLUP-EBV based on own performance (OP) or progeny testing (PT) information, in cases with a limited number of phenotypic records available to develop a reference population. We focussed on the equilibrium response obtained after a few generations of selection [13]. Thus, we first investigated the reduction of accuracy and response to selection due to the effect of selection on the genetic variance, the so-called Bulmer effect [13]. Second, we investigated the optimal construction of the reference population when the number of phenotypic records is limited. In dairy cattle, construction of a reference population started with genotyping progeny tested bulls, merely because accurate EBV based on progeny testing were available for these bulls [3]. When the number of phenotypic records is limiting, *e.g.,* when records still need to be collected, it may however be suboptimal to use progeny tested individuals to construct the reference population. Finally, we investigated the minimal size of the reference population necessary for GS to become advantageous over traditional BLUP EBV selection, and the dependency of this break-even point on heritability and generation interval.

## Methods

### Predicting response to selection

Response to selection is predicted with deterministic simulations based on selection index theory, using the SelAction software. SelAction predicts the response to selection and accuracy of selection for breeding programs. The software accounts for reduction in variance due to selection, known as the “Bulmer-effect” [13], and for the use of pedigree information, as with selection on BLUP-EBV. Features of SelAction and the theoretical background are described in [14]. Genomic selection schemes can be simulated in SelAction by including an additional trait representing the marker information [2,15]. The marker information was modelled as a trait with a heritability of 0.999, which was genetically correlated to the trait of interest. The genetic correlation between the marker information and the trait of interest was equal to the accuracy of genomic EBV, $r_{g\hat{g}}{}_{0}$, which depends on the reference population. The $r_{g\hat{g}}{}_{0}$ represents the accuracy of genomic EBV in an unselected population and is calculated using Equation 2a-d given below [6]. Because it is assumed that genotypes can be observed without error, the marker information is fully heritable and has no residual variance. Thus, the environmental correlation between the marker information and the trait of interest is meaningless and was set to zero in SelAction. Further details of this approach are given in [15].

Because the accuracy of genomic EBV established in the reference population, $r_{g\hat{g}}{}_{0}$ refers to the accuracy in a population that is not under selection, there is a distinction between $r_{g\hat{g}}{}_{0}$ and the Bulmer equilibrium accuracy of a breeding scheme based on genomic selection, denoted $r_{g\hat{g}}{}_{eq\text{.}}$ in this paper. The Bulmer effect reduces the proportion of genetic variance explained by the markers, so that $r_{g{\hat{g}}_{eq\text{.}}}$ will be smaller than $r_{g\hat{g}}{}_{0}$ in an on-going breeding scheme. The results of the deterministic simulations presented in the Results and Discussion section refer to the Bulmer-equilibrium accuracy and response that are reached after a few (≥ 3) generations of selection.

### The Bulmer effect

The deterministic simulations accounted for the Bulmer effect. Numerical results may, however, give less insight into the magnitude of the Bulmer effect than a simple mathematical expression. In this section, therefore, a mathematical expression is derived for the Bulmer-equilibrium response, accuracy and additive genetic variance with genomic selection.

In generation *t*, the variance of the genomic EBV of the *selected* parents can be expressed as

(1) $$\sigma_{M,t}^{2*}=\sigma_{M,t}^{2}(1-k)\text{,}$$

where the * denotes values after selection, *σ*_*M,t*_^2^ is the variance of the genomic EBV before selection, *i.e*. among the candidates for selection in generation *t*, and *k* is the proportional reduction in the variance of the selection criterion due to selection of the parents. Because *k* refers to the variance of the selection criterion, rather than the variance of true breeding values, it is independent of the accuracy of selection. With truncation selection on a normally distributed selection criterion, *k* is determined entirely by the intensity of selection, *k* = *i* (*ix*), where *i* is the intensity of selection and *x* the standardized truncation point [13,16-19]. Values of *k* are usually in the range of 0.7 to 0.9. To illustrate the impact of the Bulmer effect as simple as possible, equal selection intensity is assumed here for both sexes.

The variance of the genomic EBV in the next generation is the sum of the between-family variance of genomic EBV and the Mendelian sampling variance of the genomic EBV, which originates from segregation of the markers,

(2) $$\sigma_{M_{t+1}}^{2}=\frac{1}{2}\sigma_{M_{t}}^{2}(1-k)+\frac{1}{2}\sigma_{M_{0}}^{2}\text{,}$$

where the $\frac{1}{2}\sigma_{M_{0}}^{2}$ is the Mendelian-sampling variance of the genomic EBV, which is half the variance of the estimated marker effects in an unselected population. This equation assumes that the effective number of marker loci with effects is large, so that changes in marker allele frequency can be ignored in the short term. The equilibrium variance of genomic EBV follows from substituting *σ*_*Mt+*1_^2^ = *σ*_*M,t*_^2^ and solving for *σ*_*M,t*_^2^, which yields

(3) $$\sigma_{M_{eq.}}^{2}=\frac{\sigma_{M_{0}}^{2}}{1+k}\text{.}$$

For example, for a phenotypic variance of *σ*_*P*_^2^ = 1, and a heritability of the unselected base-generation of *h*_0_^2^ = 0.3, an initial accuracy of genomic EBV of $r_{g{\hat{g}}_{0}}$ = 0.8, and a selected proportion of 5%, so that *k* = 0.86, the initial variance of genomic EBV equals 0.8^2^ × 0.3 × 1 = 0.192 and the equilibrium variance of genomic EBV equals 0.192/1.86 = 0.103.

Since response (R) to GS equals *iσ*_*M*_, which is proportional to *σ*_*M*_ rather than *σ*_*M*_^2^, the equilibrium response to GS, expressed relative to initial response, equals

(4) $$\frac{R_{eq.}}{R_{0}}=\frac{1}{\sqrt{1+k}}\text{,}$$

This results shows that the relative reduction in response due to the Bulmer effect is independent of the accuracy of selection, which agrees with results from the deterministic simulations (See Results and Discussion). For example, for a selected proportion of 5%, the Bulmer effect reduces response to GS by $1-1/\sqrt{1.86}$ = 27%, irrespective of the initial accuracy of genomic EBV.

The Bulmer-equilibrium accuracy and additive genetic variance with GS can also be derived from the unselected base-generation parameters. The equilibrium additive genetic variance follows from $\sigma_{A_{eq.}}^{2}=\frac{1}{2}\sigma_{A_{eq.}}^{2}(1-k\;r_{g{\hat{g}}_{eq.}}^{2})+\frac{1}{2}\sigma_{A_{0}}^{2}$, where the first term represents the between-family variance in true breeding values, $r_{g{\hat{g}}_{eq\text{.}}}$ is the Bulmer-equilibrium accuracy of genomic EBV, and the second term is the Mendelian sampling variance of the true breeding values. Solving for $\sigma_{A_{eq.}}^{2}$ yields

(5) $$\sigma_{A_{eq.}}^{2}=\frac{\sigma_{A_{0}}^{2}}{\left(1+kr_{g{\hat{g}}_{eq.}}^{2}\right)}\text{.}$$

The Bulmer-equilibrium accuracy follows from $r_{g{\hat{g}}_{eq.}}^{2}=\sigma_{M_{eq.}}^{2}/\sigma_{A_{eq.}}^{2}$ and substitution of Equations 1a and c, which yields an expression for equilibrium accuracy in terms of base generation parameters,

(6) $$r_{g{\hat{g}}_{eq.}}=\sqrt{\frac{r_{g{\hat{g}}_{0}}^{2}}{1+k(1-r_{g{\hat{g}}_{0}}^{2})}}\text{.}$$

Continuing the above example yields an equilibrium accuracy of $\sqrt{0.8^{2}/[1+0.86(1-0.8^{2})]}$ = 0.70.

An expression for the Bulmer-equilibrium additive genetic variance, expressed in terms of base-generation parameters, follows from substitution of Equation 1d into 1c,

(7) $$\sigma_{A_{eq.}}^{2}=\sigma_{A_{0}}^{2}\left(1-\frac{k\;r_{g{\hat{g}}_{0}}^{2}}{1+k}\right)\text{.}$$

Continuing the above example yields an equilibrium additive genetic variance of 0.3[1-(0.86×0.8^2^)/(1+0.86)] = 0.21. Equations 1a through 1d show that Bulmer-equilibrium parameters can be calculated from base-generation parameters using simple equations.

The equilibrium additive genetic variance can be understood as the sum of the equilibrium variance of the genomic EBV, which is given by Equation 1a, and the variance of the prediction error, which is unaffected by selection,

(8) $$\begin{array}{l} \sigma_{A_{eq.}}^{2}=\sigma_{M_{\mathrm{eq}}}^{2}+PEV=\frac{r_{g{\hat{g}}_{0}}^{2}\sigma_{A_{0}}^{2}}{1+k}+(1-r_{g{\hat{g}}_{0}}^{2})\sigma_{A_{0}}^{2}\\ =\sigma_{A_{0}}^{2}\left(1-\frac{k\;r_{g{\hat{g}}_{0}}^{2}}{1+k}\right) \end{array}$$

### Construction of the reference population to maximise accuracy

Accuracies of genomic EBV depend on the size of the reference population (*n*_*P*_), the effective number of loci for which effects have to be estimated (*n*_*G*_), and the correlation of the true breeding value of a genotyped individual with its phenotypic record (*r*). In a random sample of the population, the accuracy of genomic EBV, $r_{g{\hat{g}}_{0}}$, being the correlation between the genomic EBV and the true breeding value, can be calculated using

(9) $$r_{g{\hat{g}}_{0}}=\sqrt{\frac{\lambda \;r^{2}}{\lambda \;r^{2}+1}}\text{,}$$

where λ = *n*_*P*_/*n*_*G*_*n*_*P*_ being the number of individuals in the reference population with both phenotypic records and genotypes [6]. Parameter *n*_*G*_ depends on the historical effective size of the unselected population (*N*_*E*_) and on the size of the genome, *L* in Morgan, and can be estimated as [20],

(10) $$n_{G}=2N_{E}L\text{.}$$

When genotyping and phenotyping the same individuals in the reference population, *r* is equal to the square root of heritability of the trait,

(11) $$r^{2}=h^{2}\text{.}$$

When the reference population is based on progeny-tested individuals, *i.e.*, when parents are genotyped while their offspring are phenotyped, *r* equals the accuracy of EBV obtained from progeny testing [21],

(12) $$r^{2}=\frac{\frac{1}{4}\;N\;h^{2}}{1+\frac{1}{4}(N-1)h^{2}}\text{,}$$

where *N* is the number of half-sib progeny on which the EBV is based. To investigate the optimal construction of the reference population, values for $r_{g{\hat{g}}_{0}}$ were compared for different numbers of progeny per sire and reference population sizes, for a fixed heritability of 0.3.

### Response of traditional versus GS breeding schemes

For the comparison of GS with selection based on traditional BLUP-EBV estimated from phenotypic information, deterministic simulations were performed with SelAction, using the approach described above. Alternative breeding schemes were compared based on the Bulmer-equilibrium response to selection. Several selection schemes were evaluated, to illustrate the general characteristics of selection on traditional BLUP-EBV versus GS. For GS, the reference population size (*n*_*P*_), and heritability (*h*^2^) were varied, to investigate the effect of these parameters on response to selection. All other parameters were kept constant across scenarios. Selection was for a single trait and in males only. To mimic the absence of selection in females, the selected proportion in females was set to 0.99.

Three scenarios were investigated:

(1) Selection on BLUP-EBV estimated from own performance information (OP).

(2) Selection on BLUP-EBV estimated from progeny information (PT).

(3) Genomic selection based on marker information on selection candidates (GS).

To investigate the benefit of genomic information on top of phenotypic information *vs.* genomic information instead of phenotypic information, the GS scheme was applied both with and without phenotypic information on the candidates for selection.

The following assumptions were made for all scenarios:

· The population had discrete generations and a fixed number of sires and dams per generation.

· There was a population of 1 000 dams per generation.

· Twenty sires were used per generation.

· Each dam produced two male and two female offspring per generation.

· In the case of progeny testing, 10 half sib progeny per sire were available in the progeny test.

· In scenarios (1), (2) and (3) with genomic information in addition to phenotypic information, full pedigree information was available for breeding value estimation as assumed in the pseudo-BLUP selection index used in SelAction [14].

· The historical effective population size was assumed to be 100 (required for Equation 1b).

· One-stage selection, with a selection proportion of 0.02 in sires and 0.99 in dams. It was assumed that, of all progeny born, 50% were not suitable as selection candidates because of health, fertility or veterinary reasons. Therefore, we used selected proportions of 99% in females and 2% in males.

In scenario (1), OP, no phenotypic information was assumed to be available on sibs of the selection candidate.

Results will be presented in two ways. First, we compare responses to selection on traditional BLUP-EBV based on own performance or progeny information with GS, where GS schemes either include or exclude phenotypic information. Second, we identify the break-even size of the reference population at which GS without phenotypic information yields the same response as selection on traditional BLUP-EBV. In this approach, we model the break-even size of the reference population as a function of the reduction in generation interval that can be achieved when implementing GS.

## Results and discussion

Results of the deterministic simulations revealed that GS schemes show a greater reduction in response due to the Bulmer-effect than schemes with selection directly based on phenotypic information (i.e. without the use of pedigree information), such as mass selection or selection on the mean of a progeny group (Table 1). This occurs because GS targets a proportion of the genetic variation with full accuracy, whereas selection based on phenotypes targets the full genetic variation with limited accuracy. As a consequence, the genetic variance used in GS, i.e*.* the variance of the sum of marker effects, is strongly reduced by selection as a result of the build-up of negative covariances between the effects of markers (under the infinitesimal model), which in turn reduces the accuracy. This can be illustrated by a comparison of mass selection with an initial heritability of 25% to GS with an initial accuracy of 0.5, for a trait with unit phenotypic variance and 5% selected (Table 1). In an unselected population, both schemes have the same accuracy of 0.5. Results of the deterministic simulations showed that the equilibrium accuracy and additive genetic variance were 0.47 and 0.21 for mass selection but 0.39 and 0.22 for GS. Consequently, equilibrium response was 14% lower than first-generation response for mass selection but 27% lower for GS. Hence, at equilibrium, mass selection yielded 118% of the response of GS. In fact, an accuracy of 0.59 prior to selection would have been required for GS to be equivalent to mass selection at equilibrium.

**Table 1:** Comparison of the Bulmer-effect for mass selection and genomic selection

**Selection methoda**	***h***^**2**^	$r_{g{\hat{g}}_{0}}$	**Equilibrium genetic variance**	**Equilibrium accuracy**	**Δ% b**
Mass selection	0.25	0.5	0.21	0.47	−14%
Genomic selection	0.25	0.5	0.22	0.39	−27%
Mass selection	0.10	0.32	0.093	0.306	−7%
Mass selection	0.50	0.71	0.367	0.651	−21%
Mass selection	-	any value	-	-	−27%

^a^Comparison of the Bulmer-effect for mass selection and genomic selection with different heritabilities (*h*^2^) and accuracies of EBV ($r_{g{\hat{g}}_{0}}$); phenotypic variance equals 1; selected proportion equals 5%; ^b^Δ% is the relative difference between the initial response and the Bulmer-equilibrium response.

Results of the deterministic simulations also revealed a second difference between GS and mass selection. With mass selection, the reduction in response due to the Bulmer effect was greater at higher accuracy (i.e. *h*^2^). With a selected proportion of 5%, for example, response to mass selection is reduced by only 7% when *h*^2^ = 0.10 but by 21% when *h*^2^ = 0.50 (Table 1). With GS, in contrast, the reduction in response due to the Bulmer effect did not depend on the accuracy of selection. With a selected proportion of 5%, the Bulmer effect always reduced response by 27% in GS schemes, irrespective of the accuracy. Again, this occurred because the estimated genetic effects used in GS are known with full accuracy, since the markers are observed.

The above results show that reduction in response due to the Bulmer effect is always larger for GS than for selection based directly on phenotypic information (e.g*.* mass selection), except when accuracy of selection approaches 100%, in which case the reduction will be the same. The theoretical results found here (Equations 1a-e) are identical to the results found by Dekkers [18,19] for selection on traditional BLUP-EBV. Hence, the impact of the Bulmer-effect on response, accuracy and additive genetic variance is the same for GS as for traditional BLUP selection. The reduction in response to selection, for example, is independent of the accuracy of selection for both GS and selection on BLUP-EBV. The above calculations of the Bulmer effect will be approximations when marker effects are updated each generation (known as “retraining”). Nevertheless, the effect of updating marker effects is expected to be small, because the additional data becoming available for retraining each generation will usually be smaller than the already existing reference population, and the change in accuracy due to adding records to the reference population shows a diminishing return relationship. The expressions derived here for the Bulmer-equilibrium response, additive genetic variance and accuracy with GS (Equations 1a-e above) are identical to those for selection on classical BLUP-EBV presented in [22,23]. This makes sense because a model with genome-wide estimated marker effects is equivalent to a mixed model with a genomic relationship matrix [24]. Hence, results for the Bulmer effect presented here are consistent with the equivalence of GS based on estimated marker effects *vs*. a mixed model with a genomic relationship matrix. However, the derivations in [22,23] have a different foundation; they rely on the property of BLUP that selection on EBV does not affect the prediction error variance of the EBV [25,26]. Hence, the agreement of our results with those in [22,23] constitutes an independent proof of the expressions derived here.

### Construction of the reference population to maximise accuracy

**Figure 1:**
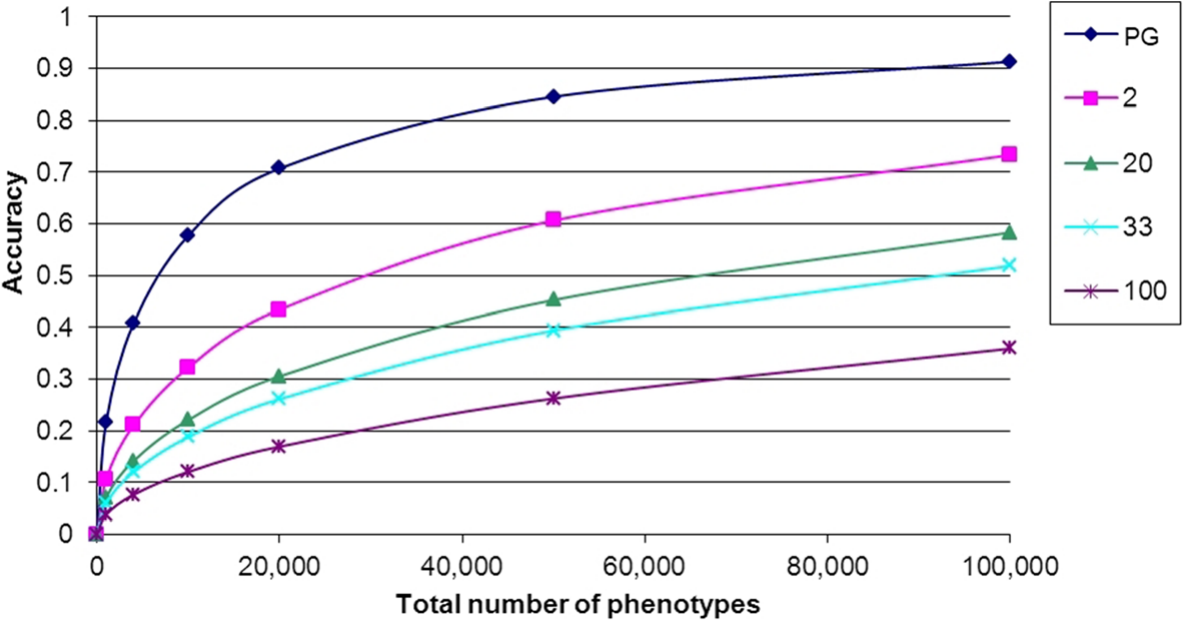
**Accuracy of genomic EBV for reference populations with different progeny group sizes.** Different lines indicate different progeny group sizes; PG = genotyped individuals in the reference population have an own performance records (same individuals phenotyped and genotyped); when the progeny group size is, e.g., 20, the number of genotyped individuals in the reference population (*n*_*P*_) equals the total number of phenotypes divided by the size of the progeny group; accuracy $r_{g\hat{g}}$ is calculated from Equations 2a-d, for N_E_ = 100, L = 30, and h^2^ = 0.3.

When the total number of phenotypic records is fixed, a reference population with phenotypic information on the genotyped individuals yields a greater accuracy of genomic EBV than a reference population with progeny information on the genotyped individuals (Equation 2, Figure 1). With progeny information, the number of sires on which the reference population is constructed will obviously decrease when the number of progeny per sire increases, which consequently reduces the accuracy, particularly when the number of phenotypic records is small (Figure 1). For example, with 4 000 phenotypic records and 20 progeny per sire, the reference population consists of *n*_*P*_ = 200 genotyped sires with EBV based on 20 progeny, whereas with two progeny per sire, the reference population consists of *n*_*P*_ = 2 000 genotyped sires with EBV based on two progeny. Although increasing the size of the progeny groups increases the accuracy of the sire’s EBV, the number of sires with which the GS reference population is constructed has a much larger impact on the accuracy of genomic EBV. Thus, when the number of phenotypic records is limiting, it is optimal to genotype the individuals that produce the phenotype, not their parents.

Studies using stochastic simulations have shown that the accuracy of genomic EBV decreases as the number of generations between the selection candidates and the animals in the reference population increases [27]. Thus, a reference population is ideally constructed using individuals most closely related to the candidates for selection [27]. Buch et al. [27] also showed that the number of daughters that need to be genotyped to replace their sires in the reference population is a function of the number of offspring underlying the sire’s EBV but is independent of the number of sires. Our results are based on a theoretical relationship between reference population size and accuracy (Equation 2), which assumes that individuals in the reference population and selection candidates are not closely related [6]. Hence, the accuracies of genomic EBV used here may be conservative. In addition, we assumed no decay of linkage disequilibrium (LD) or change in marker frequencies.

Figure 1 shows that the increase in the accuracy of genomic EBV with the number of phenotypic records is strongly non-linear, showing a diminishing-return relationship. As a consequence, increasing the total number of phenotypic records increases accuracy less than proportional. Increasing the number of phenotypes in the reference population from 5 000 to 10 000, for example, which is 2-fold, increases accuracy of genomic EBV by only 32% (Figure 1).

### Response of traditional versus GS breeding schemes

Results presented in this section refer to the Bulmer-equilibrium response and assume that the reference population is optimized for a limited number of phenotypic records. Thus, the same individuals are both phenotyped and genotyped, so that $n_{P}$ refers to both the number of phenotypic records and the number of genotyped individuals in the reference population.

**Figure 2:**
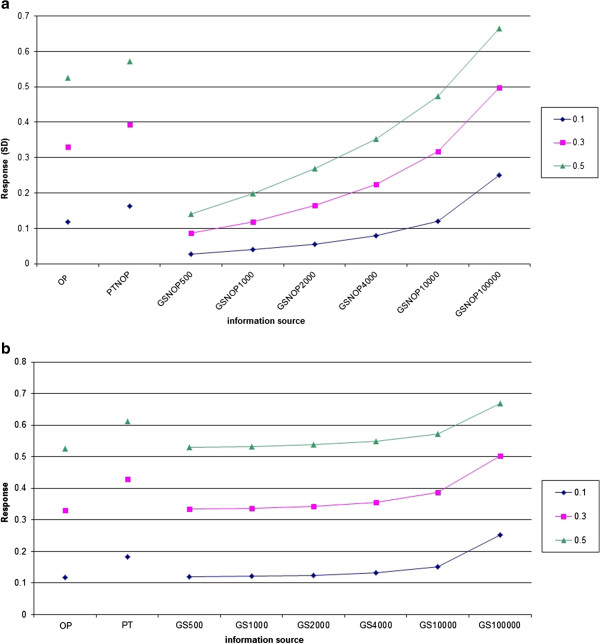
**Comparison of Bulmer-equilibrium response to selection per generation.a** - Response expressed in phenotypic standard deviations, for different information sources and reference population sizes. Different lines refer to different heritabilities; the GS schemes do not have phenotypic information on the selection candidate (NOP = No Own Performance), hence, genomic information replaces phenotypic information; OP = selection on traditional BLUP-EBV based on own performance records; PT = selection on traditional BLUP-EBV based on progeny testing; GS = genomic selection; number indicates the size of the reference population; in the reference population, the same individuals are genotyped and phenotyped; note that the x-axis scale is non-linear; for N_E_ = 100 and L = 30. **b** - Response expressed in phenotypic standard deviations, for different information sources and reference population sizes. Different lines refer to different heritabilities; in the GS schemes, individuals also have information on their own phenotype, hence, genomic information is available in addition to phenotypic information; OP = selection on traditional BLUP-EBV based on own performance records; PT = selection on traditional BLUP-EBV based on progeny testing; GS = genomic selection; number indicates the size of the reference population; in the reference population, the same individuals are genotyped and phenotyped; note that the x-axis scale is non-linear; For N_E_ = 100 and L = 30.

Figure 2a compares response to selection per generation between selection on traditional BLUP-EBV and GS schemes when the selection candidates do not have phenotypic information. The results show that large reference population sizes are required for GS to outperform traditional breeding schemes. Even with reference population sizes of 10 000 individuals, GS did not generate a larger response per generation than traditional breeding.

Figure 2b compares selection on traditional BLUP-EBV to GS schemes when the selection candidates also have phenotypic information. Hence, in the GS schemes in Figure 2b, genomic information is available in addition to phenotypic information. Results show that in these cases, GS is of little additional value, unless the reference population is very large. Figures 2a and b show that the response pattern does not change much with heritability. In conclusion, Figures 2a and b show that GS cannot compete with traditional selection when the number of phenotypic records is limited, unless the generation interval can be decreased by GS.

**Figure 3:**
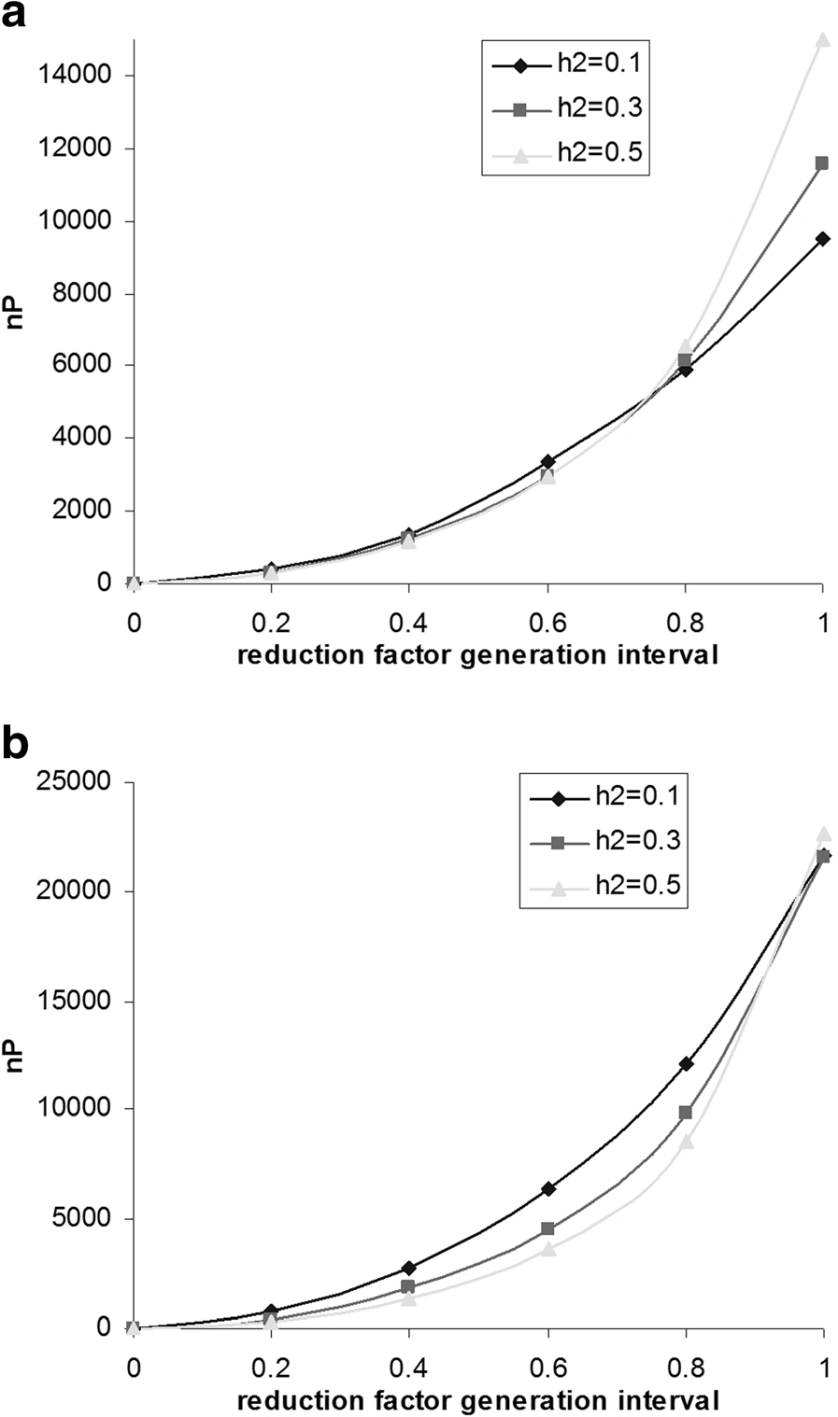
**Reference population size needed for genomic selection to reach a response per year equal to that with selection on traditional BLUP-EBV.** 3a -Based on own performance information. In the reference population, the same individuals are genotyped and phenotyped; for N_E_ = 100 and L = 30. 3b - Based on progeny-testing with 10 progeny per sire. In the reference population, the same individuals are genotyped and phenotyped; for N_E_ = 100 and L = 30.

Figures 3a and b show the break-even size of the reference population that is needed to reach a similar response to that with selection on traditional BLUP-EBV, as a function of the decrease in the generation interval that can be obtained when implementing GS. When the generation interval cannot be decreased, large reference population sizes are required, particularly when heritability is high, which agrees with Figures 2a and b. However, when generation intervals can be decreased, the break-even size of the reference population decreases rapidly, particularly when heritability is high, because of the non-linear relationship of accuracy with the number of phenotypes; a reduction in reference population size yields a less than proportional reduction in accuracy (Figure 1). In contrast, a reduction in generation interval yields a proportional increase in response. As a consequence, small reductions in generation interval lead to relatively large reductions in the break-even size of the reference population. For example, for *N*_*E*_*L* = 100 × 30, compared to selection on traditional BLUP-EBV based on own performance information, a reference population size of ~ 6 000 individuals is needed when the generation interval is reduced by 20%, whereas only ~ 2 000 individuals are needed when the generation interval is halved (Figure 3a). Compared to selection on BLUP-EBV based on 10 progeny per sire, a reference population size of ~ 10 000 individuals is needed when the generation interval is reduced by 20%, whereas ~ 3 500 individuals are needed when the generation interval is halved (Figure 3b). Because traditional progeny testing schemes require rather large numbers of phenotypic records and have long generation intervals, GS schemes will often be superior to progeny testing schemes when compared at an equal number of phenotypic records. Although precise results will depend on the value of *h*^2^ and *N*_*E*_*L*, the patterns shown in Figures 3a and b are not expected to depend on these factors.

Breeding programs usually focus on improvement of multiple traits and, thus, the generation interval is not determined by a single trait. This raises the question whether the conclusions drawn above from Figures 3a and b can be applied to breeding programs in practise. We believe they can, for the following reasons. First, for traits that are easy to record but that cannot be recorded on the selection candidates of both sexes, such as milk yield or egg number, GS is attractive since it allows a substantial reduction in generation interval. Thus, selection for such traits will not be an obstacle for the reduction in generation interval that is required to make GS of interest for traits with a limited number of phenotypic records. Second, for traits that can be recorded early in life on all candidates for selection, such as growth rate in broilers, GS can be combined with phenotypic information to estimate breeding values early in life [24]. Hence, in such cases, GS for the trait with a limited number of phenotypic records will allow a reduction in generation interval and this also increases response in traits that can be recorded on all candidates early in life. Thus, when considering multi-trait selection, GS is equally or more beneficial than suggested by results in Figures 3a and b.

In this work, the accuracy of genomic EBV was based on the expression presented in [6] (Equation 1a), rather than based on stochastic simulation. This expression is independent of allele frequencies, in contrast to the expression derived by Goddard [12]. However, Hayes et al. [20] found these two expressions to result in very similar accuracies but the method in [6] yielded slightly lower accuracies at low to moderate heritabilities. For traits with a limited number of phenotypes, heritabilities will be mostly in this low to moderate range [20]. Hence, this suggests that the accuracies used here are slightly conservative.

## Conclusions

With an equivalent intensity of selection, the reduction in response to selection due to the Bulmer-effect is the same for GS and for selection on traditional BLUP-EBV, irrespective of the accuracy of EBV used for selection. Hence, when schemes have the same selection intensity in both sexes, accounting for the Bulmer-effect is not essential to obtain the correct ranking of GS versus traditional BLUP schemes. However, when selection intensities differ between schemes, the Bulmer effect can affect the ranking and a comparison based on accuracies in an unselected population can be misleading [23]. Schemes in which selection is based directly on phenotypic information, such as mass selection, have a lower reduction in response due to the Bulmer effect than GS or traditional BLUP schemes.

To maximize the accuracy of genomic EBV when the number of phenotypic records is limiting, the same individuals should be genotyped and phenotyped, rather than genotyping parents and phenotyping their progeny. When the generation interval cannot be decreased with GS, large reference populations are required to obtain a similar response to that with own performance selection or progeny testing. However, the accuracy of genomic EBV has a diminishing-return relationship with the size of the reference population. As a consequence, when GS schemes have a moderate decrease in generation interval, relatively small reference population sizes are needed to obtain a response equal to that with selection on traditional BLUP-EBV based on own performance or progeny information. Thus, when the trait of interest cannot be recorded on the selection candidate, GS schemes are very attractive, even when the number of phenotypic records is limited, because traditional breeding schemes would have to rely on information from relatives with many phenotypic records and long generation intervals in the case of progeny testing.

## Competing interests

The authors declare that they have no competing interests.

## Authors’ contributions

EMG carried out all calculations, analysed results and wrote the manuscript. PB derived results on the Bulmer effect and assisted in drafting the manuscript. JAMA assisted in designing the research and the interpretation of the results, and provided critical reflection on the manuscript. All authors read and approved the final manuscript.
